# Treatment of bipolar depression: clinical practice vs. adherence to guidelines—data from a Bavarian drug surveillance project

**DOI:** 10.3389/fpsyt.2024.1425549

**Published:** 2024-07-02

**Authors:** Paul Kriner, Peter Brieger, Oliver Pogarell, Cornelius Schüle, Lisa Mußmann, Julie Korbmacher, Florian Seemüller

**Affiliations:** ^1^ Department of Psychiatry and Psychotherapy, kbo-Lech-Mangfall-Klinik Garmisch-Partenkirchen, Garmisch-Partenkirchen, Germany; ^2^ Department of Psychiatry and Psychotherapy, kbo-Isar-Amper-Klinikum, Haar, Germany; ^3^ Department of Psychiatry and Psychotherapy, Ludwig-Maximilians-University, Munich, Germany; ^4^ Bavarian Institute for Data, Analysis and Quality Assurance, Munich, Germany

**Keywords:** bipolar disorder, bipolar depression, guidelines, pharmacotherapy, polypharmacy, prescription rate, drug-drug interactions

## Abstract

**Objectives:**

Pharmacotherapy of bipolar depression (BPD) is confronted with major clinical challenges, like limited evidence-based treatment options, regular cases of treatment resistance, and risk of treatment-emergent affective switches. Medical guidelines can support practitioners to make decisions based on current scientific evidence. The objective of this study is to evaluate to what extent recommendations of the 2019 German S3 guidelines “Diagnosis and Treatment of Bipolar Disorders” are reflected in clinical practice in inpatient treatment.

**Methods:**

We conducted a descriptive analysis of prescription numbers in 2,627 patients with BPD in a naturalistic inpatient setting analyzing data from the ongoing Bavarian multicenter drug safety project Pharmaco-Epidemiology and Vigilance (Pharmako-EpiVig) from the years 2014–2022.

**Results:**

Of the patients, 38% were not administered any drug explicitly recommended for treatment of BPD, that is, quetiapine, lamotrigine, carbamazepine, or olanzapine. Only 6% of the patients received monotherapy with one of those drugs. Of the patients, 34% were administered ≥4 psychotropic drugs simultaneously. Patients received 912 different therapy regimens of mono or combination therapy with mood stabilizers (MS), atypical antipsychotics (AAP), and antidepressants. Of the patients, 72% received an antidepressant and 6% without concomitant prescription of an AAP or MS. Prescription rates of venlafaxine (21% to 14%) and tricyclic antidepressants (9% to 6%) decreased significantly from the first (2014–2016) to the last (2020–2022) observed time period. Of the patients, 60% received an MS. Prescription rate of valproate (22% to 14%) decreased significantly, while lithium prescription increased significantly (29% to 35%). Of the patients, 71% were administered an AAP. Quetiapine was the most prescribed drug overall (43%). Only two patients were administered a combination of olanzapine and fluoxetine.

**Conclusion:**

Our results demonstrate a substantial gap between guideline recommendations and current clinical practice. The remarkable heterogeneity in treatment regimens, with no discernible dominant treatment approach, is in part a reflection of the complexity of bipolar disorder but also substantiates the need of comprehensive recommendations regarding combination therapies. Increase in lithium prescription is an encouraging development due to its unique efficacy in maintenance treatment. To improve the quality of clinical practice guideline implementation, more randomized controlled trials should be conducted in the future to prospectively investigate different implementation strategies.

## Introduction

Bipolar disorders (BDs) are a heterogeneous group of severe affective disorders characterized by repeated, often chronic-recurrent episodes of (hypo-)mania and depression. Prevalence of psychiatric and somatic comorbidity is high. Therefore, BD not only considerably impairs psychosocial functioning but is also associated with substantial prevalence of disability and premature mortality, by suicide as well as somatic illnesses (1, 2).

In the course of illness, depressive symptoms are predominant and cause a major part of disease burden associated with BD (3, 4). Nevertheless, pharmacotherapy of bipolar depression (BPD) is still far less thoroughly investigated than pharmacotherapy of unipolar depression and major challenges, like effective prevention of suicide, insufficient response to available treatment options, controversy about the use of antidepressants (AD), and limited knowledge about combination therapies as well as subtype-specific therapy regimens remain (5–8).

An important step to overcome those challenges and improve patient outcome is the transfer of current scientific evidence in routine clinical practice. Structured assessment of complex and often contradicting research results in clinical practice guidelines can support practitioners and patients to make decisions about most appropriate care.

The German S3 guidelines “Diagnosis and Treatment of Bipolar Disorders,” first published in 2012 and updated in 2019, assess pharmacological treatment options for BPD and formulate statements about recommended and non-recommended pharmacological treatment approaches (9).

S3 guidelines are systematically developed, evidence- and consensus-based statements, generated by a representative committee in accordance with the requirements of the Association of the Scientific Medical Societies in Germany (AWMF). Resulting recommendations are graded in three categories: level A, i.e., strong recommendations that should be implemented; level B, i.e., recommendations that ought to be implemented; and level 0, i.e., open recommendations that may be considered to be implemented (10).

The pharmacological treatment algorithm for acute depression in BD, proposed by the S3 guidelines, recommends optimization of maintenance treatment, if it has already been established, as the first step. If maintenance treatment is not established, but indicated, it should be initiated. For phase-specific therapy, quetiapine, in concordance with international guidelines (11–16) is recommended as first-line treatment. In the 2019 update, it was elevated from a level B to a level A recommendation (17).

Lurasidone, in monotherapy or in combination with lithium or valproate, is the only drug with a level B recommendation. The recommendation was added to the guideline in the 2019 update. However, lurasidone is not available to physicians in Germany, since it was withdrawn from the German market in 2015, after the benefit assessment of the Institute for Quality and Efficiency in Health Care concluded that there is no proof of added benefit in the treatment of schizophrenia with lurasidone compared to other atypical antipsychotics (AAP, 18, 19).

Carbamazepine, lamotrigine, and olanzapine all have a level 0 recommendation indicating that they may be considered to be used as phase-specific treatment. Intravenous ketamine has a level 0 recommendation for treatment-resistant BPD. As non-recommended treatment options for acute BPD, the guideline lists lithium in monotherapy, valproate, aripiprazole (all level 0), and, since the 2019 update, also ziprasidone (level B) or armodafinil (level 0) in combination with a mood stabilizer (MS).

With reference to still insufficiently available data, the guideline explicitly refrains from giving a recommendation whether an AD should or should not be administered in acute depression in BD in mono or combination treatment. It also does not recommend a specific AD in regard to efficacy but emphasizes that, due to lower risk of treatment-emerging switching, a selective serotonin reuptake inhibitor (SSRI) should be preferred to venlafaxine or tricyclic antidepressants (TZA) and that bupropion should be preferred to venlafaxine (level B). It states that no recommendation can be made whether an AD or MS should be preferred in monotherapy (statement), except for patients with bipolar II disorder, where venlafaxine can be preferred to lithium (level 0). Sparse evidence for superiority of the combination olanzapine/fluoxetine to olanzapine monotherapy is mentioned. Referring to insufficient data, the guideline refrains from further recommendations about combination therapy altogether.

While clinical practice guidelines constitute a valuable source of information for practitioners as well as patients, implementation of psychiatric treatment guidelines in clinical practice has been shown to be challenging in the past, and evidence for sustainable effects of treatment recommendations on prescription practice and patient outcome is limited (20–22).

Studies have reported on inpatient prescription practice in BPD in Germany before 2010 (23, 24), since the publication of the S3 guideline; however, treatment patterns in BPD in inpatients have not been evaluated.

Therefore, the aim of this study is to assess to what extent current clinical practice reflects evidence-based recommendations of the 2019 German S3 guidelines “Diagnosis and Treatment of Bipolar Disorders.”

## Methods

### Data source

For this study, we analyzed data from the ongoing Pharmako-EpiVig project, which is collecting prescription data and reports of severe adverse drug reactions (sADR) from up to 26 psychiatric hospitals in Bavaria, Germany, since 2014. On two reference days, a year all inpatients currently being treated at the participating hospitals are included in the surveys. All drugs administered on the reference day, along with ICD-10-codes of psychiatric and somatic diagnoses, patients’ year of birth and gender, as well as all sADRs that occurred within 2 weeks before the reference day, are documented by the attending physicians and reported anonymized to the Bavarian Institute for Data, Analysis and Quality Assurance.

The study protocol and analyses have been approved by the ethics committee of the Medical Faculty of the LMU Munich.

### Study population and design

We extracted prescription data of patients with a primary diagnosis of BPD, defined as reported ICD-10-codes F31.3, F31.4, and F31.5, from the years 2014 to 2022. We did not include patients with secondary diagnosis of F31.3, F31.4, and F31.5 because we wanted to focus our analysis on patients in which BPD was the main condition treated during the hospital stay. Unfortunately, unlike DSM-V, the ICD-10 coding system does not allow to differentiate between bipolar I and bipolar II disorders. Prescription numbers and prescription rates of individual drugs and classes of drugs were assessed cumulated by calendar year and overall.

In particular, we focused on prescription of drugs from the drug classes of MSs, AAPs, and ADs, which we defined in accordance with the 2019 German S3 guidelines “Diagnosis and Treatment of Bipolar Disorders.” MSs by guideline definition comprise carbamazepine, lamotrigine, lithium, and valproate.

We assessed prevalence of monotherapies and combination therapies. Monotherapy was defined as administration of no more than one drug from the drug classes of MS, AAP, and AD and combination therapy as concurrent prescription of at least two drugs from those drug classes. We also assessed prevalence of polypharmacy, with polypharmacy being defined as concurrent administration of five drugs or more (25), as well as prevalence of complex polypsychopharmacy, which we defined as concurrent administration of four or more psychotropic drugs in concordance with previous studies (26, 27). Furthermore, we used the internet-based drug–drug interaction program mediQ to identify combination therapies with high-priority drug–drug interactions (28).

Finally, reported sADRs were searched for reports of treatment-emergent affective switches to mania or hypomania.

### Data analysis

We used the statistical software R for data analysis. Due to the naturalistic data and non-hypothesis-based nature of this study, data analysis was descriptive, and we refrained from using more elaborate statistical methods like correction for multiple testing. Data are presented as percentages for categorical variables and as means and standard deviations for continuous variables. To identify trends in prescription practice, we aggregated prescription data in three 3-year periods and used Chi-square test to test for significance of differences in prescription numbers in those time periods. Differences were reported as statistically significant if the p-value was less than or equal to 0.05.

## Results

### Study population

A total of 2,627 patients with a primary diagnosis of BPD, defined as ICD-10 diagnosis code of F31.3, F31.4, or F31.5, were included in the study. On average, 1.31 psychiatric comorbidities (SD = 0.59) were documented. Characteristics of the study population are demonstrated in **Table 1**.

**Table 1:** Characteristics of the study population.

Age (years)				
*≤30*	206 (7.8%)			
*31–60*	1,495 (56.9%)			
*>60*	923 (35.1%)			
Mean age	54.5 (SD 15.1)			
Sex				
Male	1.074 (40.9%)			
Female	1.550 (59.0%)			
Missing	3 (0.1%)			
Classification of current depressive episode by ICD-10 code				
Years	2014–2022	2014–2016	2017–2019	2020–2022
N	2,627	940	931	756
F31.3	408 (15.5%)	151 (16.1%)	150 (16.1%)	107 (14.2%)
F31.4	1,927 (73.4%)	683 (72.6%)	686 (73.7%)	558 (73.8%)
F31.5	292 (11.1%)	106 (11.3%)	95 (10.2%)	91 (12.0%)
Most frequent psychiatric comorbidities by ICD-10 code				
F10	215 (8.2%)			
F13	89 (3.4%)			
F60	76 (2.9%)			
F43	60 (2.3%)			
F17	45 (1.7%)			
F41	41 (1.6%)			
F12	40 (1.5%)			
F06	39 (1.5%)			
F45	39 (1.5%)			
F61	38 (1.4%)			

Description of the study population by age, sex, ICD-10 code of bipolar depression, and most frequent secondary psychiatric diagnoses. Psychiatric diagnoses by three-character code of International Classification of Disease in its 10th Version: F31.3: bipolar affective disorder (BD), current episode of mild or moderate depression; F31.4: BD, current episode of severe depression without psychotic symptoms; F31.5: BD, current episode of severe depression with psychotic symptoms; F10: mental and behavioral disorders due to use of alcohol; F13: mental and behavioral disorders due to the use of sedatives or hypnotics; F60: specific personality disorders; F43: reaction to severe stress and adjustment disorders; F17: mental and behavioral disorders due to the use of tobacco; F41, other anxiety disorders; F12: mental and behavioral disorders due to the use of cannabinoids; F06, other mental disorders due to brain damage and dysfunction and to physical disease; F45: somatization disorder; F61: mixed and other personality disorders.

### General prescription numbers

On average, patients were administered 5.7 drugs (SD = 3.4) simultaneously. The average number of psychotropic drugs administered was 3.1 (SD = 1.41). The average number of psychotropic drugs administered was highest in patients with diagnosis of F31.4 (3.19, SD = 1,42), followed by F31.5 (3.08, SD = 1.37) and F31.3 (2.71, SD = 1,3). Prevalence of complex polypsychopharmacy, defined as concurrent prescription of four or more psychotropic drugs was 34.4% (N = 905). Prevalence of complex polypsychopharmacy decreased significantly (χ2 = 4.08, p = 0.043) from 37.7% (N = 354) in the first (2014–2016) to 33.0% (N = 249) in the last time period (2020–2022).

A total of 45 (1.7%) patients were not administered any psychotropic drugs at all. Another 30 patients (1.1%) were treated with psychotropic drugs, but not with an MS, AAP, or AD. In this group, benzodiazepines/benzodiazepine receptor agonists (N = 23) and typical antipsychotics (N = 16) were the most prescribed psychotropic drugs.

### Mood stabilizers

Of the patients (N = 1,575), 60.0% were administered at least one MS. Prescription rate was highest in F31.3 (61.5%), followed by F31.4 (60.7%) and F31.5 (53.1%). Decline in prescription rate of valproate was more pronounced in male (26.6%, 89/335 to 16.1%, 49/304) than in female patients (19.7%, 113/575 to 12.9%, 58/450). **Table 2** demonstrates the number and proportion of patients with MS prescriptions.

**Table 2:** Prescription numbers of mood stabilizers.

	2014–2022	2014–2016	2017–2019	2020–2022	
Mood stabilizer	1,575 (60.0%)	579 (61.6%)	560 (60.2%)	436 (57.7%)	χ2 = 2.68, p = 0.10
Lithium	851 (32.4%)	271 (28.8%)	318 (34.2%)	262 (34.7%)	**χ2 = 6.60, p = 0.01**
Valproate	491 (18.7%)	202 (21.5%)	181 (19.4%)	108 (14.3%)	**χ2 = 14.56, p < 0.001**
Lamotrigine	365 (13.9%)	144 (15.3%)	118 (12.7%)	103 (13.6%)	χ2 = 0.97, p = 0.33
Carbamazepine	26 (1.0%)	13 (1.4%)	8 (0.9%)	5 (0.7%)	χ2 = 2.08, p = 0.15

Number and proportion of patients with prescription of at least one mood stabilizer defined in accordance with the 2019 German S3 guidelines for bipolar depression, and number and proportion of patients with prescription of specific mood stabilizers, overall and for 3-year time periods. The last column demonstrates the results of the Chi-square test comparing prescription rates of the first (2014–2016) and last (2020–2022) time periods. Bold font emphasizes changes in prescription over time that were significant at p ≤ 0.05.

### Atypical antipsychotics

Of the patients (N = 1,873), 71.3% were administered at least one AAP. Prescription rate was highest in F31.5 (83.9%), followed by F31.4 (70.5%) and F31.3 (65.9%). **Table 3** demonstrates the number and proportion of patients with AAP prescriptions.

**Table 3:** Prescription numbers of atypical antipsychotics.

	2014–2022	2014–2016	2017–2019	2020–2022	
Atypical antipsychotic	1,873 (71.3%)	659 (70.1%)	655 (70.4%)	559 (73.9%)	χ2 = 3.05, p = 0.08
Quetiapine	1,138 (43.3%)	389 (41.4%)	423 (45.4%)	326 (43.1%)	χ2 = 0.52, p = 0.47
Aripiprazole	376 (14.3%)	136 (14.5%)	125 (13.4%)	115 (15.2%)	χ2 = 0.18, p = 0.67
Olanzapine	335 (12.8%)	121 (12.9%)	102 (11.0%)	112 (14.8%)	χ2 = 1.33, p = 0.25
Risperidone	216 (8.2%)	75 (8.0%)	67 (7.2%)	74 (9.8%)	χ2 = 1.71, p = 0.19

Number and proportion of patients with prescription of at least one atypical antipsychotic, and number and proportion of patients with prescription of quetiapine, aripiprazole, olazapine, and risperidone, overall and for 3-year time periods. The last column demonstrates the results of the Chi-square test comparing prescription rates of the first (2014–2016) and last (2020–2022) time periods. Amisulpiride (41), clozapine (26), ziprasidone (17), paliperidone (10), sulpiride (10), asenapine (9), cariprazine (7), and benperidol (3) were each administered in less than 2% of the patients. No patient was administered lurasidone.

### Antidepressants

Of the patients (N = 1,888), 71.9% were administered at least one AD. Prescription rate was highest in F31.4 (76.7%), followed by F31.3 (59.6%) and F31.5 (57.2%). **Table 4** demonstrates the number and proportion of patients with AD prescriptions.

**Table 4:** Prescription numbers of antidepressants.

	2014–2022	2014–2016	2017–2019	2020–2022	
Antidepressant	1,888 (71.9%)	690 (73.4%)	666 (71.5%)	532 (70.4%)	χ2 = 1.92, p = 0.17
SSRI	703 (26.8%)	257 (27.3%)	241 (25.9%)	205 (27.1%)	χ2 = 0.01, p = 0.92
SNRI	676 (25.7%)	257 (27.3%)	244 (26.2%)	175 (23.1%)	**χ2 = 3.88, p = 0.049**
TZA	205 (7.9%)	88 (9.4%)	74 (8.0%)	43 (5.7%)	**χ2 = 7.93, p = 0.005**
Venlafaxine	472 (18.0%)	197 (21.0%)	168 (18.0%)	107 (14.2%)	**χ2 = 13.19, p < 0.001**
Mirtazapine	418 (15.9%)	150 (16.0%)	146 (15.7%)	122 (16.1%)	χ2 = 0.01, p = 0.92
Sertraline	412 (15.7%)	122 (13.0%)	156 (16.8%)	134 (17.7%)	**χ2 = 7.36, p = 0.007**
Bupropion	210 (8.0%)	68 (7.2%)	64 (6.9%)	78 (10.3%)	**χ2 = 5.06, p = 0.02**
Duloxetine	153 (5.8%)	55 (5.9%)	50 (5.4%)	48 (6.3%)	χ2 = 0.18, p = 0.67
Escitalopram	153 (5.8%)	60 (6.4%)	50 (5.4%)	43 (5.7%)	χ2 = 0.36, p = 0.55
Agomelatine	88 (3.3%)	36 (3.8%)	35 (3.8%)	17 (2.2%)	χ2 = 3.46, p = 0.06
Citalopram	85 (3.2%)	50 (5.3%)	22 (2.4%)	13 (1.7%)	**χ2 = 15.18, p <.001**
Amitriptyline	60 (2.3%)	24 (2.6%)	24 (2.6%)	12 (1.6%)	χ2 = 1.88, p = 0.17
Milnacipran	55 (2.1%)	5 (0.5%)	27 (2.9%)	23 (3.0%)	**χ2 = 16.26, p < 0.001**
Trimipramine	54 (2.1%)	26 (2.8%)	15 (1.6%)	13 (1.7%)	χ2 = 2.04, p = 0.15

Number and proportion of patients with prescription of at least one antidepressant, with prescription of at least one drug from the drug classes of selective serotonin reuptake inhibitor (SSRI), serotonin–norepinephrine reuptake inhibitor (SNRI), and tricyclic antidepressant (TZA) and with prescription of specific antidepressants, overall and for 3-year time periods. The last column demonstrates the results of the Chi-square test, comparing prescription rates of the first (2014–2016) and last (2020–2022) time periods. Bold font emphasizes changes in prescription over time that were significant at p ≤ 0.05. Tranylcypromine (42), trazodone (38), fluoxetine (31), opipramol (30), tianeptine (30), clomipramine (20), paroxetine (22) doxepin (16), moclobemide (16), nortriptyline (16), imipramine (12), vortioxetine (11), reboxetine (5), maprotiline (4), and fluvoxamine (1) were prescribed in less than 2% of the patients.

### Other psychotropic substances

Of the patients (N = 751), 28.6% were administered a benzodiazepine (BZD) or benzodiazepine receptor agonist (BzRA). Prescription rate of BZD/BzRA decreased significantly (p = 0.016) from 31.7% in the first time period (2014–2016) to 26.3% in the last time period (2020–2022). Among the 35 most administered psychotropic drugs were several first-generation antipsychotics. Of the patients, 7.7% (N = 201) were administered pipamperone, 2.7% (N = 71) prothipendyl, 2.2% (N = 59) melperone, 1.7% (N = 44) promethazine, 1.5% (N = 40) haloperidol, and 1.3% (N = 35) flupentixol. Pregabalin was administered in 5.4% of the patients (N = 141). Oxcarbazepine (N = 16) and pramipexole (N = 11) were administered in less than 1% of the patients. Ketamine was administered in five patients on the reference days. No patient was administered armodafinil.

### Monotherapy

Of the patients (N = 403), 15.3% were administered only one drug from the drug classes of AAPs, MSs, and ADs. The proportion of patients with monotherapy did not change significantly from 14.7% in the first time period (2014–2016) to 14.6% in the last time period (2020–2022). Patients with monotherapy were administered 32 different drugs from the drug classes of AAPs, MSs, and ADs.

Of the patients (N = 104), 4.0% received monotherapy with an MS, 2.1% (N = 56) with lithium, and 1.2% (N = 32) with valproate. Lamotrigine (N = 12) and carbamazepine (N = 4) in monotherapy were prescribed in less than 1% of the patients.

Of the patients (N = 180), 6.9% received monotherapy with an AAP, 4.3% (N = 114) with quetiapine, 1.0% (N = 27) with olanzapine. Aripiprazole (N = 16), risperidone (N = 13), amisulpride (N = 5), ziprasidone (N = 2), clozapine (N = 1), paliperidone (N = 1), and sulpiride (N = 1) in monotherapy were prescribed in less than 1% of the patients.

Of the patients (N = 119), 4.5% received monotherapy with an AD. A proportion of patients with monotherapy with an AD did not change significantly from 4.3% in the first time period (2014–2016) to 4.1% in the last time period (2020–2022). In total, 1.5% (N = 40) received monotherapy with an SNRI and 1.4% (N = 38) with an SSRI. Less than 1% of the patients received a TZA (N = 4) as monotherapy. Venlafaxine (N = 26), mirtazapine (N = 20), and sertraline (N = 19) were the most often prescribed ADs in monotherapy. An additional 1.7% of the patients were treated with two (N = 37) or three (N = 8) ADs without concurrent treatment with an AAP or MS.

### Combination therapy

Patients with combination therapy received 880 different drug regimens with up to seven drugs from the drug classes of MSs, AAPs, and ADs. Of the patients (N = 921), 35.1% were administered dual therapy, with two drugs from the drug classes of AAP, MS, and AD, and 32.4% of the patients (N = 850) were administered triple therapy with drugs from those drug classes. Of the patients (N = 307), 11.7% were administered quadruple therapy, 2.7% of the patients were administered five (N = 59), six (N = 10), or seven (N = 2) of those drugs simultaneously. In total, 27.7% of the patients (N = 728) were administered MSs, AAPs, and ADs simultaneously. Only two patients were prescribed a combination of olanzapine and fluoxetine. **Table 5** shows the most commonly administered drug regimens in the study population. **Table 6** shows the most commonly administered drug class combinations. **Table 7** shows the most commonly combined combinations of ADs, AAPs, and MSs in patients with ≥2 prescriptions of those drug classes.

**Table 5:** Most commonly administered drug regimens.

Quetiapine monotherapy	114 (4.3%)
Lithium monotherapy	56 (2.1%)
Quetiapine + lithium	67 (2.6%)
Quetiapine + sertraline	53 (2.0%)
Quetiapine + venlafaxine	45 (1.7%)
Quetiapine + valproate	37 (1.4%)
Valproate monotherapy	32 (1.2%)
Lithium + sertraline	31 (1.2%)
Quetiapine + mirtazapine	31 (1.2%)
Lithium + venlafaxine	29 (1.1%)
Olanzapine monotherapy	27 (1.0%)
Venlafaxine monotherapy	26 (1.0%)
Quetiapine + lithium + venlafaxine	26 (1.0%)
Lithium + olanzapine	23 (0.9%)
Quetiapine + lithium + sertraline	23 (0.9%)

Absolute numbers and prescription rates of the 15 most commonly administered drug regimens of antidepressants, atypical antipsychotics, and mood stabilizers.

**Table 6:** Most commonly administered combinations of drug classes.

AD + AAP + MS	422 (16.1%)
AD + AAP	354 (13.5%)
AD + MS	250 (9.5%)
AAP + MS	235 (8.9%)
AD + AD + AAP	129 (4.9%)
AD + AD + AAP + MS	121 (4.6%)
AD + AD + MS	111 (4.2%)
AD + AAP + AAP + MS	67 (2.6%)
AD + AAP + AAP	66 (2.5%)
AD + AAP + MS + MS	52 (2.0%)

Absolute numbers and prescription rates of the 10 most commonly prescribed combinations of drug classes from antidepressants (AD), atypical antipsychotics (AAP), and mood stabilizers (MS).

**Table 7:** Most commonly combined drugs.

Quetiapine + lithium	326 (12.4%)
Quetiapine + valproate	202 (7.7%)
Quetiapine + venlafaxine	202 (7.7%)
Quetiapine + sertraline	196 (7.5%)
Quetiapine + mirtazapine	181 (6.9%)
Lithium + venlafaxine	167 (6.4%)
Quetiapine +lamotrigine	144 (5.5%)
Lithium + sertraline	136 (5.2%)
Lithium + mirtazapine	135 (5.1%)
Quetiapine + aripiprazole	128 (4.9%)
Lithium + olanzapine	111 (4.2%)
Lithium + aripiprazole	94 (3.6%)
Venlafaxine + mirtazapine	94 (3.6%)
Quetiapine + bupropion	90 (3.4%)
Lithium + lamotrigine	78 (3.0%)

Absolute numbers and prescription rates of the 15 most commonly administered combinations of antidepressants, atypical antipsychotics, and mood stabilizers in patients with ≥2 prescriptions of those drug classes.

### Number of patients without prescription of guideline recommended drugs

Of the patients (N = 1,007), 38.3% did not receive any drug with an explicit recommendation for treatment of acute BPD (quetiapine, carbamazepine, lamotrigine or olanzapine).

Of the patients (N = 426), 16.2% did not receive any drug with a guideline recommendation for maintenance treatment to prevent recurrence of depressive episodes (quetiapine, carbamazepine, lamotrigine, olanzapine, lithium, or valproate).

Of the patients (N = 355), 13.5% did not receive any drug with a guideline recommendation for maintenance treatment to prevent recurrence of manic episodes (quetiapine, carbamazepine, olanzapine, lithium, valproate, aripiprazole, risperidone, or paliperidone).

Of the patients (N = 276), 10.5% did not receive any drug with a guideline recommendation for maintenance treatment (quetiapine, carbamazepine, lamotrigine, olanzapine, lithium, valproate, aripiprazole, risperidone, or paliperidone).

### High-priority drug–drug interactions

The drug–drug interaction program mediQ identified 91 drug combinations in 89 patients with high-priority drug–drug interactions. Most frequent (N = 30) were combinations of lithium with diuretics, in particular hydrochlorothiazide. Combinations of citalopram or escitalopram with other QT-prolonging drugs, like quetiapine, risperidone, or haloperidol, were also common (N = 24). Eight combinations of quetiapine with carbamazepine were identified. By induction of the metabolizing liver enzyme CYP3A4, carbamazepine can reduce bioavailability of quetiapine to approximately 15%.

Seven critical combinations of tranylcypromine with other ADs, namely, trimipramine and doxepin (serotonin toxicity), or bupropion and maprotiline (a.o. risk of seizures) were identified.

### Treatment-emergent affective switches (TEAS)

Two incidents of TEAS to hypomanic phases were reported from 2016 to 2022. In one case, duloxetine was the accused agent. The patient was also administered lithium. In response to the affective switch, duloxetine was discontinued, and the patient was started on quetiapine. In the other case, clomipramine and mirtazapine were the accused agents. The patient was also administered olanzapine. In response to the affective switch, the dose of mirtazapine was reduced, and the patient was started on lithium.

## Discussion

Our results demonstrate a substantial gap between the recommendations of the 2019 German S3 guidelines “Diagnosis and Treatment of Bipolar Disorders” and current clinical practice in the treatment of BPD. Of the patients, 38% did not receive any drug explicitly recommended for the treatment of acute BPD, namely, quetiapine, olanzapine, lamotrigine, or carbamazepine.

### Guideline-recommended treatment options

A recent network meta-analysis by Yildiz et al. (29) corroborated the guideline recommendations for quetiapine, olanzapine, and lamotrigine concluding that there is moderate evidence that they are efficacious in the treatment of BPD. Quetiapine alone was superior to placebo in reducing affective switches. Combination treatment with olanzapine/fluoxetine, which only two patients in our study population were prescribed, had the largest effect size of all included treatment options. In regard to carbamazepine, it concluded that there is no clear evidence for superiority to placebo. Other drugs with moderate evidence for efficacy were lumateperone, which is not yet approved by the European Medicines Agency (EMA), lurasidone, which has been withdrawn from the German market, and cariprazine. Cariprazine, which was EMA approved for the treatment of schizophrenia only shortly before the last update of the guideline was prescribed to only seven patients in our study population. Recent studies show promising results regarding cariprazine for augmentation in treatment-resistant BPD and in treatment of anxiety symptoms in patients with BPD (30, 31).

In accordance with guideline recommendations, quetiapine was the most prescribed drug overall. Quetiapine is also the only drug approved for treatment of acute BPD in Germany. After quetiapine was introduced in Europe, within 10 years, prescription rate increased rapidly to approximately 40% in around 2010 (23, 32). Our results demonstrate a plateau in prescription rate at approximately 43%. Quetiapine monotherapy, the only drug regimen with a level A recommendation, was the most commonly administered drug regimen, with a prescription rate of 4%. The prescription rate of olanzapine also remained stable at approximately 13%, slightly higher than in previous studies in outpatients with BD (33, 34). Greil et al. have demonstrated that in the 2000s, olanzapine prescription decreased as quetiapine prescription increased (23).

Quetiapine and olanzapine are not only recommended for treatment of acute BPD but also for phase-specific treatment of mania and maintenance treatment in BD. Common side effects of quetiapine and olanzapine include sedation and extrapyramidal side effects (35–37). Prescription of quetiapine and even more so of olanzapine is, however, mostly limited by metabolic side effects, in particular weight gain and consequent risk of metabolic syndrome (38–40). Poor adherence is also a significant challenge in patients with prescription of AAPs (41).

Of the patients, 14% were prescribed lamotrigine, which has a level 0 recommendation for the treatment of acute BPD and a level B recommendation for prophylaxis of depressive episodes in BD, though drug approval in Germany is limited to the latter. The weak level 0 recommendation is based on only two controlled studies in outpatients, one in patients with bipolar I compared to placebo (42) and the other in patients with bipolar II compared to lithium (43). Of all drugs that were found to be superior to placebo in the network meta-analysis by Yildiz et al. (29), lamotrigine had the smallest effect size. Additionally, the use of lamotrigine in the treatment of acute BPD is limited by the necessity of slow dose titration, due to the risk of Stevens–Johnson syndrome. Nevertheless, considering the generally favorable side effect profile (44), the efficacy in the prophylaxis of depressive episodes, and the positive results for lamotrigine as adjunctive treatment to lithium or quetiapine in the acute episode (45–47), it is surprising that it is not prescribed more often. Previous studies about prescription practice in the 2000s showed considerable higher prescription rates in outpatients (48), as well as in inpatients (23). In an outpatient setting, patients and practitioners might more often prioritize tolerability over efficacy leading to more frequent prescription of lamotrigine, as Hooshmand et al. proposed (48). Overall decline in prescription rate after 2010 might be caused by an increase in the prescription of quetiapine and AAPs in general, as well as negative study results about lamotrigine efficacy in acute BPD in the late 2000s (49).

Carbamazepine is recommended for the treatment of acute BPD (level 0), acute mania, and maintenance treatment. Decline in the prescription rate of carbamazepine started in the 1990s, with the emergence of alternative treatment options, like AAPs (23). The guideline recommendation, indicating that carbamazepine may be considered to be used as phase-specific treatment, is based solely on the 2007 RCT by Zhang et al. (50) In our study population, the use of carbamazepine was almost negligible presumably because of the overall questionable efficacy of carbamazepine in the treatment of acute BPD (29) and in particular because of concerns about drug–drug interactions, as well as the unfavorable side effect profile (51).

### Non-recommended treatment options

The guideline explicitly advises against the use of lithium in monotherapy for the treatment of acute BPD. It is, however, recommended for acute mania (level B) and the only drug with a level A recommendation for maintenance treatment. In our study population, 2% of the patients were prescribed lithium in monotherapy. A recent systematic review by Fountoulakis et al. (52) also concluded that efficacy of lithium in the treatment of acute BPD in mono or combination therapy is not proven, but refers to some positive results regarding lithium in combination with other agents, like pramipexole and inositol or adjunctive lamotrigine, L-sulpiride, and modafinil. Other guidelines recommend lithium as a first-line treatment in acute BPD (12, 16). The significant increase in lithium prescription rate in patients with BPD we demonstrate in the study at hand and in an earlier analysis (53) is an encouraging development due to lithium’s unique efficacy in maintenance treatment, particularly in the prevention of manic episodes (54).

The guideline also advises against the use of valproate for the treatment of acute BPD. However, it is recommended for acute mania and maintenance treatment. Yildiz et al. (29) concluded that valproate might be efficacious in the treatment of BPD, but quality of evidence is low. Other guidelines recommend valproate for the treatment of BPD (11, 12, 14).

Decline in prescription rate might be associated with decisions of the German Federal Institute for Drugs and Medical Devices (BfArM). In 2011, BfArM restricted the drug approval of valproate for the treatment of mania to patients who are not eligible for lithium treatment and drug approval for maintenance therapy to patients who have benefited from valproate treatment in acute mania (55), after a randomized, placebo-controlled trial concluded that valproate was not superior to placebo in maintenance treatment (56). In 2018, BfArM issued a Direct Healthcare Professional Communication (“Rote-Hand-Brief”) to inform healthcare professionals that for women of childbearing age, valproate, because of its teratogenicity, must only be prescribed if alternative treatments are not effective or tolerated (57). Surprisingly, we found that the decline in prescription rate was more pronounced in male than in female patients. Well after the end of our observation period, in January 2024, BfArM issued another Direct Healthcare Professional Communication, this time to warn about a potential risk increase of neurodevelopmental disorders in children, whose fathers have been treated with valproate within 3 months before conception.

Even though the guideline states that aripiprazole is not recommended for the treatment of BPD, and a recent meta-analysis by Kadakia et al. concluded that aripiprazole is not an effective treatment for acute BPD (58), it is more frequently prescribed than all recommended treatment options, but quetiapine and prescription rate have increased compared to previous studies (23). The guideline recommendation is restricted to treatment of acute mania and maintenance treatment to prevent episodes of mania. Recommendations in other guidelines are heterogenous, with most guidelines also advising against the use of aripiprazole, especially in monotherapy (59). RTCs on aripiprazole have been negative in the past (60), but some authors have pointed to methodical weaknesses, like inappropriate high dosing, in those studies (61). There is some weak evidence that aripiprazole might be effective as adjunct treatment in BPD (62). An ongoing RCT currently further investigates efficacy of aripiprazole in adjunctive treatment of BPD (63). A potential reason for the liberal prescription of aripiprazole is the benign side effect profile in regard to metabolic adverse effects compared to other AAPs (58).

In accordance with other guidelines (59),the guideline advises against adjunctive treatment with ziprasidone for BPD. Ziprasidone is recommended for the treatment of mania and as a second-line treatment for maintenance treatment in combination with valproate or lithium. Prescription of ziprasidone in our study population was negligible.

### Antidepressants

Prescription rate of ADs was approximately 10% lower than in a previous study in inpatients with BPD (23), but still substantial at 72%. With reference to still insufficient data, the guideline states that no recommendation can be made whether ADs should or should not be prescribed in acute BPD in mono or combination therapy. Results about the efficacy and safety of ADs in BPD are mixed (64). In a *post-hoc* analysis, Yildiz et al. found ADs as a drug class, in monotherapy and combination with antipsychotics, to be superior to placebo (29), while Hu et al. concluded that adjunctive treatment with ADs overall did not have a clinically significant impact on depressive symptoms (65). As Gitlin proposed (8), it will ultimately be necessary to evaluate on a more individual basis, in regard to the present subtype of the disorder (bipolar I vs. II) and specific characteristics, like presence of mixed features (66), whether ADs are a safe and effective treatment option. In particular, there are positive results for the superiority of venlafaxine over lithium in the treatment of bipolar II depression (67), and risk of affective switching also seems to be less of a risk in bipolar II depression (68).

Of the patients, 6% were prescribed ADs, without concomitant prescription of an AAP or MS, a practice other guidelines advise against (12, 16), since monotherapy with antidepressants is associated with increased risk of TEAS, in particular, treatment with TZAs (69).

Unfortunately, because of the ICD-10 coding system, it is not possible to differentiate how many of those patients have been diagnosed with bipolar I or II subtypes.

Decline in the prescription rate of venlafaxine and TZAs, and incline in the prescription rate of bupropion seem to reflect guideline recommendations, which emphasize the comparably higher risk of treatment-emerging switching associated with venlafaxine and TZAs.

The prescription rate of SSRIs remained stable. Decrease in the prescription of citalopram with a simultaneous increase in the prescription of sertraline is a trend also observed in the treatment of unipolar depression (70) and probably influenced by the Direct Healthcare Professional Communication (“Rote-Hand-Brief”) issued by BfArM in 2011, which stated the concurrent treatment with other QT-prolonging drug as a contraindication for the prescription of citalopram (71).

### Ketamine and esketamine

The guideline recommends ketamine for the treatment of treatment-resistant BPD (level 0). Since publication of the guideline, evidence for the effectiveness and safety of ketamine use in BPD has been strengthening (72–74). In our study population, only five patients were administered ketamine on the reference day. While the true number of patients being treated with ketamine is probably significantly higher, since ketamine is usually not administered more often than two times a week, considering the size of the study population, prescription numbers are still almost negligible. Esketamine, in the form of an intranasal spray, was first approved for the treatment of treatment-resistant unipolar depression by EMA in December 2019 (75). Additionally, common misconceptions about safety and tolerability of ketamine and esketamine have recently been refuted (76). Therefore, we expect that the use of ketamine and esketamine will very likely increase in the near future.

### Benzodiazepines/benzodiazepine-like drugs

Guideline recommendation for the use of benzodiazepines in BPD is restricted to short-term treatment in patients at risk of suicide (level 0). Even though benzodiazepines and benzodiazepine-like drugs are often also necessary for the management of anxiety and insomnia in patients with severe BPD, the significant trend of more restrictive prescription that we found in our study must be regarded as a positive development, since the risk of long-term use after initiation is substantial (77).

### Combination therapy

As in previous observational studies, prevalence of polypharmacy is considerable (24, 78), which substantiates the assumption that most hospitalized patients do not sufficiently benefit from monotherapy. The guideline recommends a combination treatment with MSs and AAPs for maintenance treatment if monotherapy is not sufficient, even though evidence for superior efficacy of combination treatment in maintenance treatment is scarce (79). There are no recommendations in regard to combination therapy for acute BPD. Adjunctive treatment and switching of therapy regimens are often a clinical necessity, since response and remission rates to guideline-recommended therapy, especially in case of early non-response, are unsatisfactory (80).

One in three patients in our study population was prescribed more than three psychotropic drugs simultaneously even though evidence for efficacy of extensive combination therapy to overcome treatment failure is limited (81). We found a remarkable heterogeneity in treatment of BPD. A total of 912 different mono or combination therapy regimens of MSs, AAPs, and ADs were administered, with no discernible dominant treatment approach. Part of this heterogeneity might be explained by varying preestablished maintenance treatments, psychiatric comorbidity, heterogeneous subtypes of the disorder, and the necessity to tailor side effect profiles of available drugs to individual needs.

Other reasons for extensive polypharmacy might be failure to optimize dosing of established medications or persistent prescription of ineffective medication (81).Drawbacks of polypharmacy include increased rates of side effects or non-adherence, though studies about adverse effects of polypsychopharmacy in BPD are rare (82–84).

### Drug–drug interactions

The drug–drug interaction program mediQ identified relatively few high-priority drug–drug interactions in drug regimens prescribed in our study population. Critical combinations of lithium with diuretics and quetiapine with carbamazepine can be well managed with regular therapeutic drug monitoring. Combinations of citalopram or escitalopram with other QT-prolonging drugs might require intensified ECG monitoring. A combination of tranylcypromine with serotonin reuptake inhibitors should only be considered as a last resort in patients with treatment resistance (85).

A combination of lamotrigine and sertraline was administered in 53 patients. MediQ classifies this combination as an intermediate–priority drug–drug interaction; other authors however, recommend to avoid this combination due to an increased risk of severe skin reactions and emphasize the necessity of intensified clinical monitoring (86).

### Treatment-emergent affective switches

TEAS were surely underreported. Previous studies show significantly higher incidences (68).Underreporting is most likely due to the fact that sADRs were recorded retrospectively over a period of 2 weeks before the reference day, and the data were obtained from regular patient files, not records designed to collect data for research. In one case, the TZA clomipramine and mirtazapine were the accused agents and in the other, the NSRI duloxetine. Both patients with reported TEAS to hypomanic phases were prescribed drugs with a guideline recommendation for maintenance treatment to prevent manic episodes.

### Limitations

Because of the repeated cross-sectional approach of data collection, the study design does not allow to draw conclusions about causal relationships of results. No information was available about individual treatment history, course of disease, or whether psychotropic drugs were administered for treatment of acute BPD, maintenance treatment, or concomitant psychiatric disorders.

Due to the chronic-recurrent course of disease in BD, it is likely that a considerable number of patients without guideline-recommended therapy has been treated with guideline-recommended drugs in the past and might have a history of poor response or side effects.

Since switching of medication is usually done in an overlap and taper manner, prevalence of polypharmacy is surely overestimated, and interpretation of prevalence of combination therapies is consequently limited. Additionally, psychiatric comorbidity might influence current psychotropic medications substantially.

In the period 2020–2022, patient numbers decreased approximately 20% compared to the previous years possibly due to reduced inpatient treatment capacities associated with restrictions during the COVID-19 pandemic (87). Developments in prescription rates might be influenced by differences in the study population at given reference days. Among other things, the presence of psychotic symptoms and severity of the present depressive episode influence treatment approaches. The proportions of patients with diagnosis of F31.3, F31.4, and F31.5, however, remained relatively stable over the time period. Due to the naturalistic nature of the data and exploratory approach of the analysis, we refrained from using more elaborate statistical methods, which also include correction for multiple testing. Unlike DSM-V, ICD-10 does not differentiate between bipolar I and II subtypes. It is also not possible to discern other special courses of the disorder like mixed states or rapid cycling. This limits interpretation of the data, since treatment recommendations differ in regard to subtype and special course of the disorder. Since this study analyzes data from an observational database, mis- or underreporting of diagnoses, prescription numbers, and sADRs during the process of data collection cannot be ruled out. Only inpatients were included in the study population. Comparisons to studies about prescription practice in outpatients, which, on average, suffer from less severe symptoms, are therefore only reasonable to a limited extent.

## Conclusion

Our study gives a comprehensive picture of current pharmacological treatment patterns of BPD in inpatients in Bavaria. Evidence-based treatment options for acute BPD, recommended by the guideline, are few, and each have inherent limitations regarding efficacy and tolerability. Current guideline recommendations do not seem to sufficiently meet clinical needs in inpatient treatment.

The heterogeneity in prescription practice that we found in our study suggests that in the absence of more comprehensive guideline recommendations, clinicians base their decision making on their individual clinical experience and, despite lack of evidence for the effectiveness and safety of that approach, regularly resort to complex polypharmacy to overcome treatment failure.

Inclusion of a more comprehensive treatment algorithm in the guideline, like that proposed in the CINP guideline (Table 5), (11), might be a helpful to tool to improve evidence-based clinical care, but ultimately, the heterogeneity in prescription practice is mostly a consequence of the relatively weak evidence base for all recommended drugs, except quetiapine.

Persistently high prescription rates of drugs with questionable efficacy, like ADs, corroborate the necessity for innovations in the pharmacotherapy of BPD. Treatment options, like lurasidone, the drug with the second highest recommendation level after quetiapine, and lumateperone, should be made available to the German market. In light of recent evidence, some treatment options, like olanzapine + fluoxetine, ketamine, esketamine, or cariprazine, seem to be underutilized.

Prescription practice remained relatively constant over the observed time period; increase in the prescription of lithium and bupropion and decline in the prescription of venlafaxine and TZAs reflect guideline recommendations.

In the past, numerous studies have been conducted to identify barriers to the implementation of clinical practice guidelines. However, there remains a significant lack of randomized controlled trials that scientifically assess and compare the effectiveness of various implementation methods. Soon, an interesting cluster-randomized trial implementing schizophrenia guidelines will be available (88). Such prospective studies could better inform decision makers and leaders about implementation strategies. In parallel, the degree of guideline implementation needs to be continuously observed using naturalistic datasets.

## Data availability statement

The data that support the findings of this study are available from the Bavarian Institute for Data, Analysis and Quality Assurance (BIDAQ), Am Moosfeld 13, 81829 München, E-Mail: kontakt@bidaq.de, but restrictions apply to the availability of these data, which were used under license for the current study, and so are not publicly available. Data are, however, available from the authors upon reasonable request and with permission from the Bavarian Institute for Data, Analysis and Quality Assurance (BIDAQ). Requests to access these datasets should be directed to kontakt@bidaq.de.

## Ethics statement

The studies involving humans were approved by Ethics committee of the Medical Faculty of the LMU Munich. The studies were conducted in accordance with the local legislation and institutional requirements. Written informed consent for participation was not required from the participants or the participants’ legal guardians/next of kin in accordance with the national legislation and institutional requirements.

## Author contributions

PK: Writing – review & editing, Writing – original draft, Methodology, Investigation, Formal Analysis, Conceptualization. PB: Writing – review & editing, Methodology, Formal Analysis, Conceptualization. OP: Writing – review & editing, Supervision, Conceptualization. CS: Writing – review & editing, Supervision, Conceptualization. LM: Writing – review & editing, Project administration, Methodology, Formal Analysis, Data curation. JK: Writing – review & editing, Project administration, Methodology, Formal Analysis, Data curation. FS: Writing – review & editing, Supervision, Methodology, Investigation, Conceptualization.
